# The Endogenous Inhibitor of CETP, apoC1, Remains Ineffective In Vivo after Correction of Hyperglycemia in People with Type 1 Diabetes

**DOI:** 10.3390/metabo14090487

**Published:** 2024-09-07

**Authors:** Alexia Rouland, Thomas Gautier, Damien Denimal, Laurence Duvillard, Isabelle Simoneau, David Rageot, Bruno Vergès, Benjamin Bouillet

**Affiliations:** 1 Department of Endocrinology, Diabetology and Nutrition, Dijon Bourgogne University Hospital, 21000 Dijon, France; alexia.rouland@chu-dijon.fr (A.R.); isabelle.simoneau@chu-dijon.fr (I.S.); bruno.verges@chu-dijon.fr (B.V.); 2 INSERM Research Center U1231 CTM, University of Burgundy, 21000 Dijon, France; thomas.gautier@u-bourgogne.fr (T.G.); damien.denimal@chu-dijon.fr (D.D.); laurence.duvillard@chu-dijon.fr (L.D.); david.rageot@u-bourgogne.fr (D.R.); 3 Department of Clinical Biochemistry, Dijon Bourgogne University Hospital, 21000 Dijon, France

**Keywords:** type 1 diabetes, hyperglycemia, apolipoprotein C1, CETP, glycation

## Abstract

ApolipoproteinC1 (apoC1) is the main physiological inhibitor of the cholesterol ester transfer protein (CETP). Increased CETP activity is associated with macrovascular complications in patients with type 1 diabetes (T1D). ApoC1 has lost its ability to inhibit CETP in patients with T1D, and in vitro glycation of apoC1 increases CETP activity, suggesting that hyperglycemia could be a factor implicated in the loss of the inhibitory effect of apoC1 on CETP. Thus, we aimed to see whether improvement of glycemic control might restore apoC1 inhibitory effect on CETP. We studied 98 patients with T1D and HbA1c > 9% at baseline and 3 months after improvement of glycemic control by a medical intervention (insulin introduction or changes in multi-injection therapy or pump therapy introduction/therapeutic education for all patients). CETP activity was assessed by a radioactive method and plasma apoC1 levels were measured by ELISA. The different isoforms of apoC1 were determined by mass spectrometry. CETP activity was not significantly modified after improvement of glycemic control, despite a significant reduction in mean HbA1c (8.7 ± 1.7 vs. 10.8 ± 2, *p* < 0.0001). No association between plasma apoC1 and CETP activity was observed in patients with T1D at baseline, nor at 3 months, even in the subgroup of patients with optimal control (3-month HbA1c < 7%). We did not find any glycated form of apoC1 using mass spectrometry in people with T1D. Hyperglycemia in vivo does not seem to be a major factor implicated in the loss of apoC1 ability to inhibit CETP activity observed in T1D. Other factors, such as qualitative abnormalities of lipoproteins, could be involved. Our data emphasize the fact that hyperglycemia is not the only factor involved in lipid abnormalities and macrovascular complications in T1D. Clinical trial reg. no. NCT02816099 ClinicalTrials.gov.

## 1. Introduction

ApolipoproteinC1 (apoC1) is an apolipoprotein composed of 57 aminoacids that is involved in lipoprotein metabolism [1]. ApoC1 is mostly located on high-density lipoprotein (HDL) (80%) but can also be present on the surface of very-low-density lipoprotein (VLDL) (20%) [2,3]. The biological effects of apoC1 are multiple, particularly because it is easily exchangeable [3], depending on its location on lipoproteins [4]. ApoC1 is likely to have an important role in regulating lipid metabolism. Indeed, HDL-associated apoC1 has been demonstrated to be the main physiological cholesterol ester transfer protein (CETP) inhibitor [5].

CETP is a plasmatic glycoprotein that is essential for the metabolism of HDL and triglyceride-rich lipoproteins through the promotion of cholesterol ester and triglyceride exchanges between these two lipoproteins [6,7]. CETP is associated with an increased transfer of cholesterol esters from HDL to VLDL, intermediate-density lipoprotein (IDL) and low-density lipoprotein (LDL), in vitro and in vivo, in transgenic mice expressing CETP [8,9]. This unfavorable effect of CETP on cholesterol esters brought up the idea of a pro-atherogenic role of CETP. Indeed, genome-wide association studies have shown that individuals with a genetically determined lower level of CETP mass and CETP activity have significantly lower risk of cardiovascular diseases [10,11]. A higher level of CETP was also associated with macrovascular complications in patients with type 1 diabetes (T1D) [12].

The electrostatic charge of HDL is one of the mechanisms involved in the inhibitory effect of apoC1 on CETP. Indeed, apoC1 lowers the electronegativity of HDL, which results in a dissociation of CETP from HDL [13]. The inhibitory effect of apoC1 on CETP activity has been confirmed in humans, as negative correlations were found between apoC1 and CETP activity in normolipidemic subjects [14,15,16]. However, our group previously showed that high levels of triglycerides led to a loss of positive correlation between apoC1 and CETP activity, suggesting that high levels of triglycerides could stimulate CETP activity and overwhelm the inhibitory action of apoC1 on CETP [16].

Patients with T1D usually show increased CETP activity compared to control individuals [16]. The reasons for this increase in CETP activity are still unclear, and different mechanisms may be suspected, such as chronic hyperglycemia or qualitative abnormalities of lipoproteins [17]. Glycation, a consequence of hyperglycemia, is suspected to be involved in the apoC1 inability to inhibit CETP. Indeed, the in vitro glycation of apoC1 modifies its electrostatic properties and impairs its ability to inhibit CETP, and the electrostatic properties of apoC1 were found to be modified in patients with T1D [16]. Thus, we hypothesized that hyperglycemia could be a factor implicated in the loss of the inhibitory effect of apoC1 on CETP observed in people with T1D.

The elucidation of the mechanisms underlying the loss of the capacity of apoC1 to inhibit CETP in people with T1D is of major interest because the restoration of apoC1 functionality might help to counteract the deleterious effects of increased CETP activity on the plasma lipoprotein profile in this high cardiovascular risk population. 

In order to ascertain whether hyperglycemia could be involved in the loss of the capacity of apoC1 to inhibit CETP in people with T1D, we performed a prospective study in patients with uncontrolled T1D, aiming to determine whether a significant improvement of glycemic control could restore the inhibitory effect of apoC1 on CETP.

## 2. Material and Methods

### 2.1. Study Population

This monocentric prospective study was carried out in the Endocrinology department of the University Hospital of Dijon, France. This clinical trial was registered (Clinical trial reg. no. NCT02816099 ClinicalTrials.gov) and approved by our local Ethics Committee. Written informed consent was obtained from all subjects involved in the study. Experimental protocols and process for obtaining informed consent were approved by the appropriate institutional review committee.

Ninety-eight patients with T1D (mean age = 32.8 ± 12.2 years; 54% were male) were included (Table 1). Type 1 diabetes was defined by age at diagnosis <35 years, positive islet autoantibodies and insulin requirement from the moment of diagnosis. No patient with latent autoimmune diabetes (LADA) were included in the study. We decided to include only T1D patients in order to avoid confounding factors that could influence CETP activity, such as hypertriglyceridemia, which is much more frequent in patients with type 2 diabetes than in those with T1D. At inclusion, all patients had uncontrolled T1D, with HbA1c > 9%. Patients with estrogen or long-course steroid treatment or undergoing an immunosuppressive therapy were not included. Since part of the patients were treated with statins, this treatment was taken into account in our statistical analysis.

This longitudinal study was performed as follows. During the baseline visit, general information was obtained (diabetes complications, duration, treatments against diabetes or other, smoking status, other diseases) and a physical examination was performed (weight, height, body mass index (BMI), blood pressure, sensitivity in the lower limbs, peripheral pulses). Fasting blood samples were collected during this baseline visit and a standard medical intervention was initiated to improve glycemic control. Indeed, all patients received therapeutic education. Insulin was introduced into patients with newly discovered T1D. Changes in multiple-injection therapy or switch to pump therapy was performed in other patients.

Three months later, a second visit was performed to collect another fasting blood sample.

### 2.2. Plasma Preparation

Blood samples were collected in fasting state using BD Vacutainer tubes (Becton Dickinson, Franklin Lakes, NJ, USA) with or without EDTA as the anticoagulant and preservative. They were centrifugated for serum separation within 2 h after collection. The plasma was divided into aliquots and stored at −80 °C until biological analysis. 

### 2.3. Routine Analytical Procedures

HbA1c, creatinine, microalbuminuria, fasting lipid profile were assessed during the first visit. HbA1c, creatinine and fasting lipid profile were assessed during the second visit.

Creatinine, microalbuminuria and lipid profile were determined using a Dimension Vista analyzer with dedicated reagents (Siemens Healthcare Diagnostics, Deerfield, IL, USA). HbA1c was measured with a G8 HPLC analyzer (Tosoh Bioscience, Tokyo, Japan).

### 2.4. Measurement of apoC1 and CETP Concentration by Immunoassay

Plasma apoC1 and CETP concentrations were measured using specific ELISA assays with an anti-human apoC1 antibody (Thermofisher EHAPOC1, Waltham, MA, USA) and an anti-human CETP antibody (MyBioSource, MBS2511733, San Diego, CA, USA), respectively, at baseline and 3 months later.

### 2.5. Measurement of CETP Activity Using a Radioactivity Method

Plasma CETP activity was measured using a radioactive method, as previously described [18].

Briefly, the CETP activity was evaluated as the transferred radiolabeled cholesteryl esters from [^3^H]CE-HDL to unlabeled endogenous LDL acceptor, after the subtraction of blank values.

Human HDL was biosynthetically labeled using a method described in the literature [19].

Plasma samples (25 µL) were incubated 3 h at 37 °C with 2 µL of [^3^H]CE-HDL (cholesterol concentration: 0.491 g/L). After incubation, the reaction was stopped by adding 1 mL of a potassium bromide solution (density, 1.07). Donor and acceptor lipoprotein fractions were then separated by ultracentrifugation (3 h, 100,000 rpm) in a TL100 rotor (Beckman, Palo Alto, Brea, CA, USA). Infranatant and supernatant were then transferred into counting vials filled with 2 mL of scintillation fluid. Radioactivity was measured for 2 min using a Hidex 300SL counter (Lablogic, Europa View, Tinsley, Sheffield, UK).

CETP activity was calculated as the percent of total radioactivity transferred to supernatant after subtraction of blank values obtained with plasma incubated at 4 °C. Finally, the results were normalized with the CETP mass, to be expressed as CETP activity.

### 2.6. Detection of apoC1 in Mass Spectrometry

Electrophoresis/protein extraction/in-gel digestion: The procedure we followed was described in a previous study [20].

MALDI-TOF analysis: Samples were analyzed using MALDI TOF/TOF MS (XTrem, BrukerDaltonics, Billerica, MA, USA). Calibration was performed using BTS Bacterial Test standard (BRUKER, Billerica, MA, USA) containing insulin, ubiquitin I, cytochrome C and myoglobin, for a mass range of 4000–30,000 Da, and a peptide calibration standard kit (BrukerDaltonics, Billerica, MA, USA) containing bradykinins 1–7, angiotensin I, angiotensin II, substance P, bombesin, adrenocorticotropic hormone (clips 1–17 and 18–39) and somatostatin for a mass range of 800–4000 Da. Each sample was diluted 1/50 in 0.1% TFA. 1 µL of peptide extract was mixed with 1 µL of matrix solution (10 mg HCCA 50%ACN/50%H20, 0.1% TFA for 1 mL). 1 µL from this mix was then filled in a MALDI spot metering target.

Data on MALDI TOF were acquired with reflector mode, with a 25 kV accelerating voltage, 26.3 kV reflector voltage, and 20 ns pulsed ion extraction time. 

A total of 600 to 1200 laser shots were used to obtain mass spectrum, which was then processed with Flexanalysis.

### 2.7. Measurement of Triglycerides in HDL

Samples of plasma from each patient with T1D at baseline and at 3 months were used. An amount of 100 µL of plasma were mixed with 40 µL of PEG 6000 at 20% in order to obtain apolipoprotein-B depleted plasma. After 20 min of rest at room temperature, tubes were centrifugated 30 min at 10,000 rpm. Supernatants were then collected and triglycerides were measured using Dimension Vista analyzer with dedicated reagents (Siemens Healthcare Diagnostics, Deerfield, IL, USA).

### 2.8. Statistics

Data were reported as means ± SD or percentages as indicated. The comparison between patients with T1D at inclusion and at 3 months was performed using the non-parametric paired Wilcoxon test. Univariate analyses were performed using a regression model with a robust variance estimator to evaluate factors influencing CETP activity. Statistical analyses were performed with STATA software version 14.0 (StataCorp LP, College Station, TX, USA). *p* values <0.05 were considered statistically significant.

## 3. Results

### 3.1. Participants Characteristics

**Figure 1:**
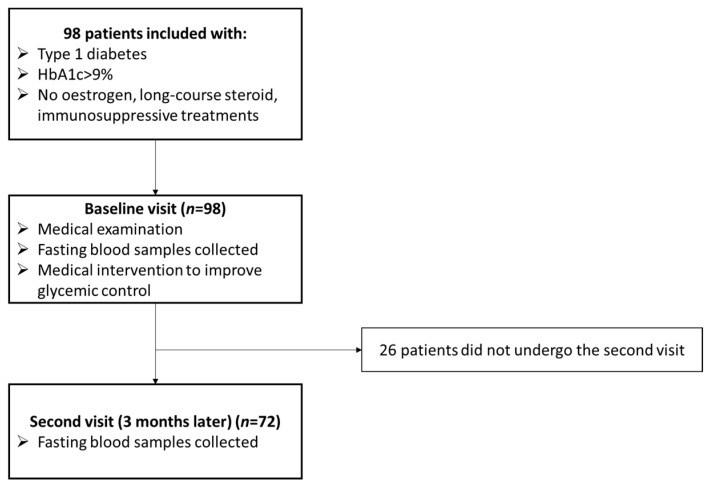
Flowchart.

Ninety-eight participants were included in the study. Twenty-six patients did not undergo the second visit. Thus, data were analyzed in 98 participants at baseline and in 72 participants at 3 months (Figure 1).

Clinical and biological characteristics of the population are summarized in Table 1. At baseline, the mean age was 35.5 ± 12.1 years and 54% of participants were male. HbA1c was significantly lower at 3 months after medical intervention (8.7 ± 1.7 vs. 10.8 ± 2%, *p* < 0.0001). BMI was 24.5 ± 5.3 kg/m^2^ at baseline and was significantly higher 3 months later (25.2 ± 4.8 vs. 24.5 ± 5.3 kg/m^2^, *p* = 0.0001).

The plasma lipid profile of T1D patients was improved after 3 months of treatment, especially with a significant increase in HDL cholesterol (1.6 ± 0.4 vs. 1.4 ± 0.4 mmol/L, *p* < 0.0001). Three months of treatment also led to significant decrease in plasma triglycerides (1.0 ± 0.5 vs. 0.8 ± 0.3 mmol/L, *p* = 0.0152) in patients with T1D, although triglycerides remained in the normal range throughout the study.

No difference in plasma apoC1 and CETP mass concentrations were observed between baseline and at 3 months. CETP activity was not significantly different in patients with T1D between baseline and at 3 months.

To better assess the influence of CETP activity after glycemic control improvement, we evaluated the triglyceride content of HDL in patients at baseline and at 3 months. HDL triglyceride content was not significantly different between baseline and at 3 months (0.162 ± 0.170 vs. 0.141 ± 0.112 mmol/L, *p* = 0.99), even when adjusted on apoAI level (0.164 ± 0.180 vs. 0.130 ± 0.105 mmol/L, *p* = 0.63).

### 3.2. Association between CETP Activity and apoC1

**Figure 2:**
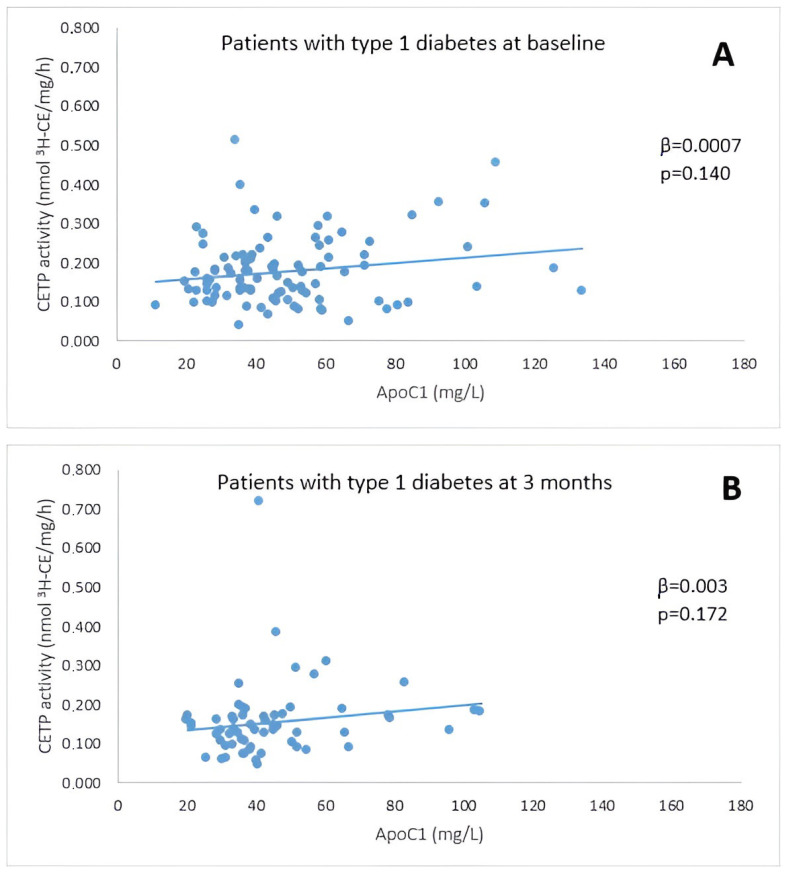
Correlation between CETP activity and apoC1 in T1D patients at baseline (**A**) and at 3 months (**B**). CETP: cholesteryl ester transfer protein.

In people with T1D at baseline, CETP activity was not associated with apoC1 (β = 0.0007, *p* = 0.140). It was, however, significantly associated with triglycerides (β = −0.043, *p* = 0.001), LDL cholesterol (β = 0.027, *p* = 0.021) and total cholesterol (β = 0.026, *p* = 0.007) (Table 2, Figure 2A).

In people with T1D at 3 months, CETP activity was not associated with apoC1 (β = 0.0028, *p* = 0.172) and it was no longer associated with any of the selected parameters (Table 2, Figure 2B).

In a subgroup of people with T1D and HbA1c < 7% at 3 months, no negative association between apoC1 concentration and CETP activity was observed.

### 3.3. Detection of apoC1 in Mass Spectrometry

**Figure 3:**
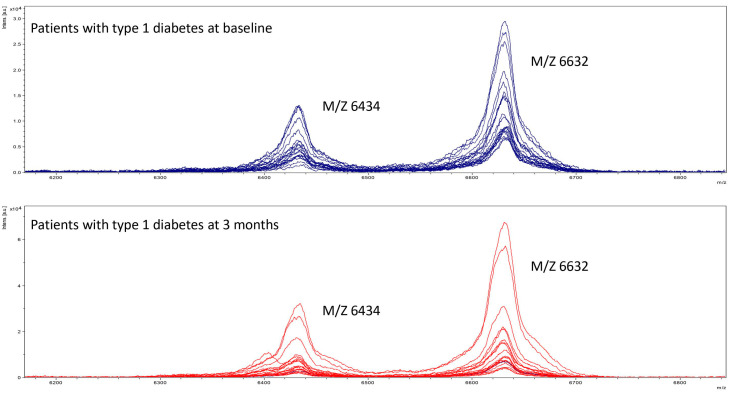
Mass spectrometry profile of apoC1 in patients at baseline and at 3 months.

ApoC1 from a subgroup of 20 patients with T1D at baseline and at 3 months was analyzed in mass spectrometry. Mass spectrometry was used to assess different isoforms of apoC1 in each group. The two peaks coincided with two known isoforms of apoC1: apoC1 (M/Z 6632) and truncated apoC1 (M/Z 6434). No third peak, that is to say, no different isoform of apoC1 was observed in patients with T1D at baseline (Figure 3). The same profile was observed in patients with T1D after improvement of glycemic control at 3 months. Thus, these data indicate the absence of any glycated form of apoC1 in patients with T1D.

## 4. Discussion

In our study, no negative association between apoC1 concentration and CETP activity was observed in people with T1D after improvement of glycemic control, suggesting that hyperglycemia does not seem to be a critical factor in vivo, explaining the loss of the capacity of apoC1 to inhibit CETP activity in people with T1D.

In the literature, CETP is independently and negatively associated with apoC1 in non-diabetic subjects [21,22]. In people with T1D, in our study, we did not observe any negative association between CETP activity and apoC1 at baseline. This statement is in line with previous works showing that apoC1 loses its ability to inhibit CETP activity in this population [16].

However, after 3 months, while glycemic control was significantly improved, we did not observe a negative correlation between the apoC1 concentration and CETP activity. Thus, the decrease in HbA1c that we observed was not accompanied by a restoration of the apoC1 inhibitory potential in our population.

The reduction in HbA1c from 10.8 to 8.7% was statistically significant. Even if this drop may seem modest in absolute values, it is clinically significant. Nevertheless, it is important to note that, despite significant glycemic improvement following medical intervention, the mean HbA1c was still 8.7% at 3 months in our population of T1D patients. In order to explore in greater detail the effect of effective glycemic control on apoC1 and CETP function, we decided to evaluate the association between CETP activity and apoC1 concentration in a subgroup of people with T1D and optimal glycemic control, which was assessed by HbA1c < 7% at 3 months (*n* = 18). No negative and significant association between CETP activity and apoC1 was observed in this population. This reinforces the fact that improving hyperglycemia does not restore apoC1 function.

These data suggest that hyperglycemia is not likely to be involved in the loss of the inhibitory effect of apoC1 on CETP. This finding is in contrast to previous results suggesting that glycation could be involved in the loss of apoC1 function observed in patients with T1D. Indeed, previous in vitro glycation experiments of apoC1 resulted in a change in its electrostatic properties and an increase in CETP activity, indicating that glycated apoC1 was no longer able to inhibit CETP activity [16]. In the present study, we aimed to expand these conclusions into the clinical situation by assessing apoC1 glycation in patients with T1D and testing whether the glycation of apoC1 could be influenced by glycemic control. The present study did not reveal any glycated form of apoC1 using mass spectrometry in samples from people with T1D, suggesting that the loss of apoC1 function observed in people with T1D is not explained by the glycation of apoC1.

Moreover, the lack of difference in apoC1 isoforms in mass spectrometry between uncontrolled T1D patients at baseline and improved glycemic control at 3 months reinforces the idea that hyperglycemia, via glycation, does not play a major role in vivo in the loss of function observed in T1D.

It is important to underline that the in vitro glycation of apoC1 in the previous study was performed with methylglyoxal [16], which does not correspond to pathophysiological conditions.

It has been shown that HDL particles are dysfunctional in people with T1D [23] due to qualitative modifications [17]. These structural and compositional alterations could explain why apoC1, which effectively inhibit CETP as an HDL component in healthy subjects, fails to inhibit CETP activity in modified HDL. Indeed, it was shown that the strength of apoC1 interaction with lipid/water interfaces depends on lipid composition, especially the phospholipid and triglyceride content [24,25]. The modified interactions between apoC1 and HDL particles might well influence apoC1 conformation and the availability of the positively charged C-terminal, lysine-rich CETP inhibitory sites [26,27].

For illustration, in the present study, we determined the triglyceride content in HDL particles and showed that it was not modified after improvement of glycemic control, suggesting that some qualitative abnormalities, such as triglyceride enrichment, which remains present after improvement of glycemic control [17], could be a factor involved in the loss of the inhibitory effect of apoC1 on CETP in T1D. This is in line with previous studies showing that several qualitative and functional abnormalities of lipoproteins are not influenced by glycemic control in patients with T1D [28,29,30]. Moreover, the stable triglyceride content of HDL after improvement of glycemic control is another piece of evidence, indirectly this time, that CETP activity is not modified after a reduction in hyperglycemia.

ApoC1 is effective as a CETP inhibitor when present at the surface of HDL. Another hypothesis that could explain the loss of function of apoC1 could be the presence of apoC1 at the surface of VLDL and no longer at the surface of HDL. When present at the surface of VLDL, apoC1 is no longer effective as a CETP inhibitor [31]. Further studies are needed to assess this question of the distribution of apoC1 at the surface of lipoproteins in people with T1D.

Our data emphasize the fact that hyperglycemia is not the only factor involved in lipid abnormalities as previously reported in several studies [17]. Moreover, an increased cardiovascular risk is observed in T1D patients with good glycemic control [32,33,34], suggesting that other factors, including lipid qualitative and functional abnormalities, could be involved [17,34].

We acknowledge that our study has some limitations. Firstly, the BMI and weight were a little higher at 3 months compared to baseline in patients. However, the BMI was included in statistical analysis and was not associated with CETP activity at baseline nor at 3 months. Thus, it seems unlikely to have modified our results. Secondly, some patients were lost to follow-up between the first and the second visit. Thus, the number of patients at 3 months was lower. However, because we included a sufficient group of patients with T1D at baseline, we remain confident in our results and think that the loss of some patients during follow-up is unlikely to have significantly affected our data. Thirdly, some patients were treated with statins. However, this treatment was not changed during the study and CETP activity was not associated with statin treatment. HbA1c remains high at 3 months (8.7%), whereas we would have expected a more significant decrease. However, this decrease (−2.1%) is statistically significant, and so the consequence on CETP activity would have been highlighted if it was observed. One may think that the absence of correlation between apoC1 and CETP could be due to the small sample size of our study. However, we have been able to perform the complete study in 72 patients with T1D, representing a significant number for a clinical study. We did not observe, in our 72 patients, a significant correlation between apoC1 and CETP activity, with *p* values of 0.140 and 0.172 at baseline and after 3 months of intensive treatment, respectively, which is quite far from 0.05. We think that the results would certainly have been similar with a larger sample of patients. Fourthly, we have no direct evidence of the lack of the inhibitory effect of isolated apoC1 on CETP in our study. However, it has been shown that purified human apoC1 inhibited CETP activity in a concentration-dependent manner [14]. In the same study, the plasma concentration of apoC1 correlated negatively with CETP activity in the same patients. This negative correlation between plasma apoC1 and CETP activity has been confirmed in other studies in normolipidemic subjects [15,16]. Thus, we considered that no correlation between plasma apoC1 and CETP activity is an indirect indicator of the loss of inhibitory effect of apoC1 on CETP.

## 5. Conclusions

Data from our study indicate that hyperglycemia does not seem to be a major factor implicated in the loss in the ability of apoC1 to inhibit CETP activity observed in T1D. Other factors, such as qualitative abnormalities of lipoproteins, could be involved in the loss of apoC1 function observed in T1D. Further studies are needed to better understand apoC1 dysfunction in T1D, which may have deleterious effects on the plasma lipoprotein profile in this high cardiovascular risk population.

## Figures and Tables

**Table 1:** Characteristics of the populations.

	Normal Range of Laboratory	T1D Patients at Baseline (*n* = 98)	T1D Patients 3 Months Later (*n* = 72)	*p*-Value (Baseline vs. 3 Months Later)
Age (years)		32.8 ± 12.2	33.8 ± 12.5	N/A
Male sex (*n*(%))		53 (54)	35 (48.6)	N/A
Weight (kg)		71.5 ± 15.8	72.5 ± 14.9	*p* = 0.0002
BMI (kg/m^2^)		24.5 ± 5.3	25.2 ± 4.8	*p* = 0.0001
Diabetes duration (years)		13.3 ± 12	14.1 ± 12.6	N/A
Diabetes complications:				
Retinopathy (*n*(%))		27 (27.6)	19 (26.4)	N/A
Nephropathy (*n*(%))		21 (21.4)	19 (26.4)	N/A
Neuropathy (*n*(%))		17 (17.3)	10 (14.5)	N/A
HbA1c (%, mmol/mol)		10.8 ± 2	8.7 ± 1.7	*p* < 0.0001
Smoking status: Smokers (*n*(%))		43 (43.9)	24 (33.3)	
Use of statins (*n*(%))		15 (15.3)	11 (15.3)	N/A
Fasting glycemia (mmol/L)	4.3–5.9	9.6 ± 4.6		
Creatinine (µmol/L)	59–104	63.6 ± 16	64.6 ± 16.2	*p* = 0.32
Total cholesterol (mmol/L)	3.1–5.7	4.7 ± 1	4.6 ± 0.8	*p* = 0.80
HDL cholesterol (mmol/L)	1.04–1.55	1.4 ± 0.4	1.6 ± 0.4	*p* < 0.0001
LDL cholesterol (mmol/L)		2.7 ± 0.9	2.6 ± 0.7	*p* = 0.22
Triglycerides (mmol/L)	0.50–1.70	1.2 ± 0.7	1 ± 0.5	*p* = 0.0002
ApoC1 (mg/L)		49.2 ± 23.2	45.6 ± 19.2	*p* = 0.85
CETP mass (mg/L)		4.9 ± 1.4	5.6 ± 2.7	*p* = 0.218
CETP activity (nmol ^3^H-CE/mg/h)		0.176 ± 0.087	0.167 ± 0.170	*p* = 0.057

BMI: body mass index, CETP: cholesteryl ester transfer protein, N/A: not appropriated.

**Table 2:** Univariate analysis between CETP activity and several parameters in T1D patients. BMI: body mass index. CETP: cholesteryl ester transfer protein.

	At Baseline		At 3 Months	
	CETP Activity		CETP Activity	
	β	*p*	β	*p*
ApoC1	0.0007	0.140	0.003	0.172
Age	0.001	0.132	0.0004	0.733
Sex	−0.0002	0.991	−0.035	0.394
Smoking status	0.001	0.938	0.022	0.681
BMI	0.001	0.566	0.008	0.234
Creatinine	0.0002	0.697	−0.0005	0.637
Triglycerides	0.043	0.001	0.167	0.125
HDL cholesterol	−0.029	0.150	−0.077	0.256
LDL cholesterol	0.027	0.021	0.122	0.095
Total cholesterol	0.026	0.007	0.086	0.162
Retinopathy	0.027	0.134	0.004	0.902
Nephropathy	0.028	0.130	0.011	0.752
Neuropathy	0.037	0.137	0.027	0.381
Diabetes duration	0.001	0.008	−0.0004	0.830
Statin treatment	0.02	0.163	0.0047	0.898
HbA1c	0.002	0.450	0.018	0.335

## Data Availability

The datasets generated during and/or analyzed during the current study are available from the corresponding author on reasonable request.
